# Data extraction from electronic health records (EHRs) for quality measurement of the physical therapy process: comparison between EHR data and survey data

**DOI:** 10.1186/s12911-016-0382-4

**Published:** 2016-11-08

**Authors:** Marijn Scholte, Simone A. van Dulmen, Catherina W. M. Neeleman-Van der Steen, Philip J. van der Wees, Maria W. G. Nijhuis-van der Sanden, Jozé Braspenning

**Affiliations:** 1 Radboud Institute for Health Sciences, Scientific Institute for Quality of Healthcare, Radboud University Medical Center, Geert Grooteplein 21, 6525 EZ Nijmegen, The Netherlands; 2 Present address: Faculty of Social Sciences, Department of Sociology, Radboud University, Thomas van Aquinostraat 6, 6525 GD Nijmegen, The Netherlands; 3 Research Institute for Health Sciences, Scientific Institute for Quality of Healthcare, Radboud University Medical Center, Nijmegen, The Netherlands; 4 ROS Caransscoop, Beekbergen, The Netherlands

**Keywords:** Electronic health records, Data quality, Completeness, Correctness, Data sources, Healthcare quality indicators, Physical therapy

## Abstract

**Background:**

With the emergence of the electronic health records (EHRs) as a pervasive healthcare information technology, new opportunities and challenges for use of clinical data for quality measurements arise with respect to data quality, data availability and comparability. The objective of this study is to test whether data extracted from electronic health records (EHRs) was of comparable quality as survey data for the calculation of quality indicators.

**Methods:**

Data from surveys describing patient cases and filled out by physiotherapists in 2009-2010 were used to calculate scores on eight quality indicators (QIs) to measure the quality of physiotherapy care. In 2011, data was extracted directly from EHRs. The data collection methods were evaluated for comparability. EHR data was compared to survey data on completeness and correctness.

**Results:**

Five of the eight QIs could be extracted from the EHRs. Three were omitted from the indicator set, as they proved too difficult to be extracted from the EHRs. Another QI proved incomparable due to errors in the extraction software of some of the EHRs. Three out of four comparable QIs performed better (*p* < 0.001) in EHR data on completeness. EHR data also proved to be correct; the relative change in indicator scores between EHR and survey data were small (<5 %) in three out of four QIs.

**Conclusion:**

Data quality of EHRs was sufficient to be used for the calculation of QIs, although comparability to survey data was problematic. Standardization is needed, not only to be able to compare different data collection methods properly, but also to compare between practices with different EHRs. EHRs have the option to administrate narrative data, but natural language processing tools are needed to quantify these text boxes. Such development, can narrow the comparability gap between scoring QIs based on EHR data and based on survey data.

EHRs have the potential to provide real time feedback to professionals and quality measurements for research, but more effort is needed to create unambiguous and uniform information and to unlock written text in a standardized manner.

**Electronic supplementary material:**

The online version of this article (doi:10.1186/s12911-016-0382-4) contains supplementary material, which is available to authorized users.

## Background

Quality measurement is becoming an integral part of healthcare systems. With the emergence of the electronic health records (EHRs) as a pervasive healthcare information technology, [1] new opportunities and challenges for use of clinical data arise with respect to data quality, data availability and comparability [2]. However, to support the use of EHR data for quality measurements over the use of conventional data sources such as administrative data, patient surveys or provider surveys, a stronger evidence base is needed with respect to data attributes relevant for these measurements [3–5]. Comparative research between EHR data and conventional data sources, in particular provider surveys, is scarce however. This study compares data from a provider survey to calculate eight QIs that measure the quality of the physical therapy care process with data extracted directly from EHRs, thereby contributing to the scarce comparative knowledge of the use of EHR data for quality measurements.

The information in most EHRs can be distinguished in structured coded data and unstructured narrative data [3, 6]. To decrease the registration burden, most quality measures are restricted to the structured coded data. However, coded data is by definition limited in the amount of information it contains. For the medical professional, textboxes are preferred to codes, since patient information is typically easier described in a narrative manner. These text fields however are difficult to incorporate in quality measures, other than establishing whether the text box was used. A physician reporting retrospectively on patient cases in a survey can answer the questions with all of the available patient information in the back of his mind. It can be argued that physician-reported survey data represent a more holistic view on the quality of care delivered. When extracting loose chunks of information from EHRs for quality measurements such a full picture of the patient case is not possible, although the risk of bias is smaller. Also, it is questionable whether all data one can retrieve from survey items can be extracted from EHRs. A survey is specifically designed to measure the quality of care, whereas most EHRs are developed for much broader purposes, such as administration, reporting and clinical reasoning. Extracting the right pieces of information from the EHRs to be able to calculate QI scores is a technological challenge. The differences between using EHR data and survey data for quality research as well as limitations and benefits of both data collection methods (see Table 1. for examples) provide ample opportunities for comparative research between these two methods. In a recent review on methods and dimensions of quality assessment of EHR data, out of 95 reviewed articles 57 conducted comparative research of which only nine compared EHR data to survey data or patient interviews [7]. In a more recent review describing the reliability and validity of EHR data, 35 studies were reviewed, of which only four compared EHR data quality to survey data [3]. Different data attributes or dimensions of quality were studied with a great variety in terms used to describe those data attributes [3, 4]. In most studies, completeness (i.e. the level of missing data) and accuracy (or correctness compared to a gold standard) of the data was examined [3]. Chan et al. acknowledged another dimension of quality assessment which is data comparability, i.e. similarity in data quality and availability of measurement components in different data sources [3]. The importance of data comparability within the EHRs itself for reliable and valid quality measurement comparisons has been previously recognized [8, 9].

**Table 1:** (Dis)advantages of quality measurements using survey data and EHR data

Survey data	EHR data
• Sample data • Time investment for respondents • Survey gives professional more time to reflect on patient case • Possible selection and recall bias • Standardized data	• Continuous dataflow • No extra time investment • Under documenting leads to incompleteness • Minimizes bias through direct data extraction from EHRs • Differences in EHR software suppliers might lead to differences in output

In the Netherlands, a unique possibility arose for comparative research between the use of EHR data and survey data for quality measurements. The project “quality of physical therapy care” (Qualiphy) started in 2009 with the collection of survey data to calculate QI scores (see Additional file 1). In 2011, existing EHRs were adapted so that data for quality measurement of the domain physical therapy care process could be extracted directly in order to calculate QI scores. The conversion process from using survey data to EHR data to measure the quality of care and the consequences of this conversion on the quality of the data could therefore be studied in detail. Through studying the transition process from survey data to EHR data, we were able to answer whether it is possible to calculate QIs from EHR data in such a way that it leads to comparable QI scores. Comparability of the data quality must be assessed with respect to completeness and correctness. These data attributes affect the reliability and validity of the data and thus of the quality of care measurements [3, 7, 10].

Our research question therefore is: ‘*To what extent is data extracted from EHRs comparable to survey data with respect to content and data quality for scoring QIs’*?

## Methods

### Study population, data collection and quality indicators

We conducted a prospective cohort study with three cohorts (2009, 2010 and 2011). Physical therapists from around 7,200 physiotherapy practices in primary care were invited in 2009 by the Royal Dutch Society for Physical Therapy (KNGF) to participate in a program to evaluate the quality of the physical therapy care process based on eight quality indicators (see Additional file 1). Data for the quality domain physical therapy care process were retrieved from provider surveys in 2009 and 2010. Each therapist was asked to complete at least 30 surveys, scoring the physical therapy process as described in the (paper) patient files. In 2009 participation was completely voluntary. Practices that did not participate in 2009 were urged to do so by the KNGF and health insurers in 2010. In 2011, from April to June, EHRs were adapted and an extraction algorithm was constructed so that the data needed to calculate the indicators could be extracted directly. There were 15 different EHR suppliers and two third (68.4 %) of the participating practices used one of the two largest EHR suppliers. 92.5 % of participating practices used an EHR from one of the five largest suppliers. Participation in 2011 was mandatory to be eligible for contracts with health insurers. The data was collected from August to November of each cohort and contained items about the process and outcomes of physical therapy care. To compare the data quality of the two methods, only physical therapists that had participated in both the survey cohort (2009/2010) and in the EHR cohort (2011) remained in this study sample.

The original quality indicators and the items are based on guidelines that addressed the registration of the clinical reasoning process [10] and were tested on validity and reliability [11]. The indicator set contained eight indicators for the quality of the physical therapy care process, one for every step in the clinical reasoning process. Most of the indicators consisted of multiple items (see Table 2).

**Table 2:** Original quality indicators for physical therapy care process: short description, definition of indicators and items measured

Indicator no.	Short description	Indicator definition	Item measured in questionnaire
1	Direct access: Screening- and diagnostic process	The average degree (in %) in which the direct access patients received a methodically performed screening and diagnostic process (5 items)	*Request for help*: 1. asked, and 2. administrated
			*Conclusion screening*: 3. administrated
			*Diagnosis*: 4. determined systematically, and 5. administrated
2	Referred patients: Diagnostic process	The average degree (in %) in which referred patients received a methodically performed diagnostic process (4 items)	*Request for help*: 1. asked, and 2. Administrated *Diagnosis*: 3. determined systematically, and 4. administrated
3	Intervention goals	The average degree (in %) in which intervention goals were determined methodically for the patients (4 items)	*Goal(s)* 1. defined, and 2. Administrated 3. fitted to request for help 4. based on diagnosis
4	Intervention process	The average degree (in %) in which patients received a methodically performed intervention process (5 items)	*Goal(s):* 1. defined (see ind 3 item 1), and 2. administrated (see ind 3 item 2) 3. reached (main goal) *Intervention(s):* 4. Administrated *Intervention result(s):* 5. administrated (see 5)
5	Administration intervention results	The percentage of patients whose notes record the intervention results (1 item)	Note in record
6	Perceived intervention results	The average degree (in %) in which the intervention goals (total recovery, reduction of complaints or stabilization) in terms of function and activity and participation are considered to be reached for the patient (max. 15 items)	Perceived result per goal (maximum of 15 goals) response: not at all, somewhat, largely, completely
7	Measured intervention results	The average degree (in %) in which the intervention goals (total recovery, reduction of complaints or stabilization) in terms of function and activity and participation have been reached by use of measurement instruments (max. 15 items)	Result of objective measure per goal (maximum of 15 goals) response: not at all, somewhat, largely, completely
8	Information shared and agreed with the patient	The average degree (in %) in which information was shared with and agreed upon by patients (7 items)	*Shared and agreement information on:* 1. Screening process direct access patient
			2. Diagnostic process
			3. Defined goals
			4. Intervention process
			5. (Interim) evaluation
			6. Outcomes
			7. Closure of episode

Because we used de-identified data, our study was deemed exempt from review by the Medical Ethical Committee Arnhem and Nijmegen. The study was conducted in accordance of the Declaration of Helsinki.

### Extraction of data from EHRs

EHRs for physical therapy already existed, mainly for administrative purposes. The project on measuring quality of performance highlighted the desire to expand the EHR function to administrate reporting on clinical reasoning in order to be able to extract process and outcome data to compute quality indicators. In a focus group of physical therapists, software suppliers, health insurers and researchers, the quality indicators and survey items were assessed for their suitability for extraction from the EHRs and for technical feasibility. To support the data extraction and comparability of the output, a uniform extraction algorithm was constructed and supplied to all EHR software suppliers.

Throughout the process of making the EHRs available for data extraction, we complied with legislative requirements to ensure the privacy and anonymity of the patients.

### Data analysis

Descriptive statistics were used to present characteristics of the patients, physiotherapists and physiotherapy practices in both data sets. *Completeness* was defined as ‘the proportion of patient cases without missing values at item level’. To calculate *completeness,* the number of patient cases per therapist that did not have any missing values on one of the items of an indicator (nominator) was divided by the total number of patient cases per therapists on that indicator (denominator) leading to a proportional score between 0 and 1 for each indicator. The survey data was then matched at therapist level with the EHR data to compare completeness. *Correctness* was assessed by comparing the mean indicator scores per indicator in the EHR data to the survey data, which we considered to be the benchmark, in the absence of a separate gold standard. As we are matching the indicator scores of therapists calculated from survey data to the indicator scores of that same therapist calculated from EHR data, the scores should match if the data collection method was of no influence. It should therefore provide evidence whether the EHR data is an accurate representation of the quality of care provided by the therapists. To test the statistical significance of differences between survey and EHR data, a Wilcoxon matched pairs signed rank test for non-parametric data was used. This test is suitable for dependent sample comparison of ordinal variables with a skewed distribution and tests whether the median difference is zero.

The data was analyzed using SPSS version 20. Statistical significance in all analyses was determined at a *p*-value of 0.001. A relative change of 5 percent between survey and EHR data was considered to be relevant based on consensus in the project team.

## Results

### Patients and practices

A total of 5,960 physical therapists of around 2,400 practices provided data in both the provider survey as well as through data extraction directly from EHRs, describing the physical therapy process of around 160,000 patient cases in the survey and around 90,000 patient cases in the EHRs (see Table 3). Compared to a national representative sample, patient characteristics were largely representative, except for the percentage of patients with chronic diseases or conditions [12]. Patients with chronic diseases or conditions are underrepresented in the EHR data. Not all patient characteristics could be extracted correctly from the EHRs. Gender and age of the patient were only extracted in half of the patient cases. Therapist characteristics are representative with respect to age and gender [13]. The number of solo practitioners in both the survey as in the EHR data was underrepresented, and the larger practices were overrepresented [14].

**Table 3:** Characteristics from the participating practices and patients in comparison to representative samples

	Survey data		EHR data		National representative samples 2010	
	%	N	%	N	%	N
*Patient characteristics*						
Male	42.2	164,090	44.3	45,408	39.3^12^	9,301
Direct access patients	38.3	164,164	44.0	86,282	46.9^12^	9,301
Age categories						
Age 0-14	6.4	164,090	3.4	45,012	2.6^12^	9,301
Age 15-24	8.7	164,090	9.6	45,012	9.8^12^	9,301
Age 25-44	27.8	164,090	26.6	45,012	23.5^12^	9,301
Age 45-64	35.7	164,090	37.1	45,012	27.5^12^	9,301
Age 65 and older	21.3	164,090	23.3	45,012	26.6^12^	9,301
Chronic^a^	16.0	152,796	8.9	86,282	16.0^12^	9,301
Total N		164,164		86,282		
*Therapist characteristics*						
Male	47.9	5,960	49.0	5,938	45.2^13^	16,521
Age (mean)	44.3	5,727	44.3	5,706	42^13^	16,521
Total N		5,960		5,960		
*Practice characteristics*						
No of therapists per practice						
1	3.2	1,934	3.2	1,929	30.7^13^	4,770
2	18.8	1,934	18.7	1,929	14.8^13^	4,770
3-4	33.6	1,934	34.0	1,929	22.9^13^	4,770
5+	44.5	1,934	44.2	1,929	31.6^13^	4,770
Mono disciplinary	71.4	2,351	71.3	2,431	61^14^	1,969
Multidisciplinary	28.6	2,351	28.7	2,431	39^14^	1,969
Total N		2,356		2,440		

^a^Chronic patients are defined as having treatment episodes of 3 months or more

### Extraction of data from EHRs

In the transition from using survey data to using EHR data for quality measurements, decisions were made by the focus group that affected the quality indicator set at three levels. First, it was established which indicators could be extracted from the EHRs. Second, the definition and calculation of the quality indicators were modified and last, changes at item level were made.

As a result of the discussion in the focus group to make the EHRs suitable to extract data for the quality assessment, a decision was made which of the eight original quality indicators (see Table 2) could be successfully extracted from the EHRs. The experts decided to omit indicator 4 (clinical reasoning during the intervention process; defining and administrating intervention goals, interventions, and intervention results) and indicator 7 (measured intervention results by the use of measurement instruments), to combine indicator 5 (intervention results administered) and indicator 6 (perceived intervention results) and to extract a simpler form of indicator 8 (information shared with patient) out of the EHRs. Indicator 4 would not be included in the set to be extracted from the EHRs, because of the narrative character of this registration. Experts within the focus group objected to the limited list of treatment interventions to choose from. Also, some EHR software suppliers already had their own standard list of treatment interventions, while most of them used a text field. These differences turned out to be insurmountable on short notice, resulting in removal of the indicator. Indicator 7 was not included as not all recommendations in the guidelines required the use of measurement instruments. The experts from the focus group concluded that it was therefore not a valuable proxy for quality of care, given that the quality indicators were meant to be generic so that broad comparisons could be made at the level of physical therapists, as opposed to specific indicators that could compare quality at the level of the patient (or the condition of the patient). It also proved too difficult in the short term to match all possible conditions of the patients with the measurement instruments. Last, indicator 8 would be incorporated in the EHRs in a simpler form. This indicator was already part of a patient survey and it was deemed redundant to ask the professional in such an elaborate way.

Secondly, it was decided that the definition of the quality indicators should become stricter. The indicators calculated from the survey data were defined as ‘the degree in which the steps in clinical reasoning were followed’. As there turned out to be a high level of ceiling effects (therapists with a maximum mean score on an indicator) [11], it was decided that the definition of the quality indicators should be dichotomized: either the physical therapist followed all the steps in clinical reasoning, for example with regard to the screening and diagnostic processes, or he or she did not. Such a change affected the calculation of the indicators and therefore its comparability. For example; an indicator was calculated from 2 items in the survey. Item 1 had two answer possibilities (yes = 1/no = 0) and item 2 had three answer possibilities (no = 0, somewhat = 1, completely = 2). The indicator score would then be calculated as followed: actual score/maximum possible score. If a therapist scored item 1 with ‘yes’ (1) and item 2 with ‘somewhat’ (1), his score actual score for that patient case would be 2. Divided by the maximum possible score (3), the indicator score would be 2/3 = 0.67. So this proportional score would mean that the therapist followed the clinical steps for indicator 1 for 66.7 %. A mean score of all the patient case scores would then be calculated to reach the indicator score at therapist level.

In the EHR however, the item scores and the indicator scores were dichotomous instead of proportional. The definition was changed to ‘followed all the steps in clinical reasoning’ for each indicator. Both items in our example would now have a 0-1 scoring possibility and only when the physical therapist scored a patient case with the value 1 on both items, the indicator score for that patient case would be 1. At therapist level, the definition for the quality indicators would now be ‘the proportion of patient cases in which the therapist followed all of the steps in clinical reasoning.

Given the differences in definition and calculation between the survey data and the EHR data, they cannot be compared as is. The only way to compare them properly is to recode the survey items into dichotomous items, and recalculate the indicator scores in the same way as was done in 2011, the last level of change. In our example, item 1 would remain the same, but item 2 had to be dichotomized. The category ‘no’ would be recoded into 0, whereas the categories ‘somewhat’ and ‘completely’ would be recoded into 1. In the EHR data it was not possible to distinct ‘somewhat’ from ‘completely’, as it could only be assessed whether the question was answered, instead of the degree in which the question was answered. Therefore, these categories were combined. The indicator score from the survey would not be 0.67, but 1. If the therapist described 10 patient cases and he followed all the steps in clinical reasoning for that indicator in eight of them, his proportional indicator score would be 0.8.

Despite the uniform extraction algorithm, two of the largest EHR suppliers deviated from this algorithm. Because of these errors, we were unable to extract the correct data from their EHRs for indicator 5 (Intervention result). In the end, only 11.6 percent of all patient cases in the EHR data had a valid score on this indicator. It was therefore decided that this indicator could not be compared in this study as the reliability on this indicator was too low.

The final result of the transition from using survey data to using EHR data was that survey data could be compared to EHR data with respect to completeness and correctness on four indicators; screening and diagnosis for self-referred patients (indicator 1) and for referred patients (indicator 2), goal setting (indicator 3), and information shared with and agreed upon by the patient (indicator 8).

### Completeness

The EHR data showed to be significantly different (*p* < 0.001) compared to survey data on all four indicators with respect to completeness (Table 4). Completeness of EHR data is significantly higher on two of the compared indicators (indicators 1 and 8) with a relative change of more than 5 %. The improvement on indicator 8 (information shared with and agreed upon by the patient) is the largest (relative change = 217.5 %). Completeness of indicator 2 (screening and diagnosis of referred patients) is 8 percent lower for EHR data (*p* < 0.001). Although correctness of indicator 3 (goal setting) is also significantly higher in the EHR data (*p* < 0.001), the relative change is less than 5 % (4.1 %). Overall, the completeness is above 90 percent for all indicators in both survey data and EHR data.

**Table 4:** Related samples Wilcoxon Signed Rank test for *completeness* ^*a*^ of survey data and EHR data

Indicator	Survey data	EHR data			
	(%)	(%)	% relative change^b^	Z-score	N
1 – Screening and diagnostics direct access patient	92.2	99.9	+8.4	35.6*	4,583
2 – Screening and diagnostics referred patient	99.9	91.9	-8.0	-20.6*	5,565
3 – Main goal administrated	92.4	96.2	+4.1	28.8*	5,840
8 – Information shared with patient	31.4	99.7	+217.5	66.2*	5,860

^*a*^In % physical therapists without missing values

^b^Measured as (EHR-Survey)/Survey*100 %

*p < 0.001

### Correctness

Although the indicator scores of the EHR data are significantly different (*p* < 0.001) from the indicator scores of the survey data (Table 5), only the difference in indicator 1 (screening and diagnosis for self referred patients) is above the 5 percent threshold of relevant difference with a decrease in indicator score of 8.4 percent in the EHR data. Indicator 1 and 2 (screening and diagnosis) show a lower indicator score in the EHR data, while indicators 3 (goal setting) and 8 (information shared with and agreed upon by patients) have slightly higher indicator scores in the EHR data when compared to the survey data.

**Table 5:** Related samples Wilcoxon Signed Rank test for *correctness* of indicator scores^a^ of survey data and EHR data

Indicator	Survey data	EHR data			
	Mean score (sd)	Mean score (sd)	% relative change^b^	Z-score	N
1 – Screening and diagnostics direct access patient	0.97(0.11)	0.90(0.26)	-7.2	-16.17*	4,553
2 – Screening and diagnostics referred patient	0.99(0.05)	0.95(0.16)	-4.0	-13.15*	5,121
3 – Main goal administrated	0.96(0.12)	0.99(0.06)	+3.1	24.61*	5,818
8 – Information shared with patient	0.85(0.27)	0.87(0.24)	+2.4	4.055*	5,602

^a^Mean score for survey and EHR data, ranging from 0-1

^b^Measured as (EHR-Survey)/Survey*100 %

*p < 0.001

In Table 6 an overview of the results is presented.

**Table 6:** Overview of results on comparability of QIs, QI scores and results on completeness and correctness of EHR data compared to survey data^d^

	Changes from survey data to EHR data						Results	
Indica-tor	Extracted from EHR	Definition/calculation indicator changed	Items recoded	Original survey score	Recalculated survey score	EHR score	Comple-teness EHR data^b^	Correct-ness EHR data^c^
QI 1	Yes	Yes	Yes	0.90	0.97	0.89	+	-
QI 2	Yes	Yes	Yes	0.86	0.99	0.94	-	+
QI 3	Yes	Yes	No	0.95	0.96	0.99	+	+
QI 4	No	n.a.	n.a.	0.91	n.a.	n.a.	n.a.	n.a.
QI 5^a^	No	Yes	n.a.	0.95	n.a.	n.a.	n.a.	n.a.
QI 6^a^	No	Yes	n.a.	0.96	n.a.	n.a.	n.a.	n.a.
QI 7	No	n.a.	n.a.	0.78	n.a.	n.a.	n.a.	n.a.
QI 8	Yes	Yes	No	0.87	0.85	0.84	+	+

^a^Indicator 5 and 6 were to be combined when extracted from EHR

^b^+ EHR data is more complete; - EHR data is less complete

^c^+ QI scores are the same (relative change < 5 %); - QI scores are not the same (relative change > 5 %)

^d^n.a. not applicable

## Discussion

Our study showed that changes in data collection methods from survey data to data extracted from EHRs had a major impact on the comparability of the content. Survey data had to be recalculated to fit the redefined quality indicators from the EHR data. Further, only four out of eight indicators could be compared as three indicators were discarded in the transition from survey to EHR data and a fourth was not comparable due to errors in the software of two of the largest EHR suppliers, which blocked extraction of the correct data. The data quality of the indicators that we could compare showed that the EHR data was more complete than survey data on three out of four indicators and indicators based on EHR data seemed to be as accurate or correct as the indicators based on survey data on three out of four indicators.

### Explanations for the findings

Chan et al. concluded that comparability, both of EHR data to other data sources, as well as comparability between EHRs was of importance to valid care quality comparisons and outcome research [3]. Differences in what data elements needed for a measure are present in the data sources, but also variation in EHR content, structure and data format or extraction procedures can significantly affect data comparability. Due to major changes needed for the extraction of data from EHRs and time pressure, it was decided that not all quality indicators could be extracted from the EHRs, limiting the comparison of the entire quality indicator set. Ambiguous and inconsistent operationalisations of two of the largest EHR software providers caused errors in data extraction that further limited comparability. A pilot phase was initially planned in the project to test the extraction procedure with similar patient cases. However, due to enormous pressure from different stakeholders for a rapid implementation, the pilot phase was skipped with all its consequences. Despite the presence of a uniform extraction algorithm, two of the largest software providers deviated from this algorithm, blocking extraction of the correct data. An important indicator for patient outcomes (the combination of indicator 5 (result administrated) and indicator 6 (subjective result)) could therefore not be compared. Not only is the outcome of a treatment an important measurement of the quality of care, this indicator also showed the largest variation in an earlier study evaluating the psychometrical properties of the quality indicators in the survey data [11]. It could therefore have been of great interest for comparisons. In the end, four out of the original eight indicators were comparable.

One of the benefits of using EHRs is that it serves as a tool to facilitate completeness of administering the medical process. We found evidence for this because there were less missing values in the EHR data than in the survey data for three out of four quality indicators. Indicator 2 (screening and diagnosis for referred patients) had slightly more missing values in the EHR data. This could be caused by the fact that as the patients are referred, some of the steps in the clinical reasoning process were already performed by the referring physician and simply not administrated properly in the EHR by the treating physical therapist. Further, indicator 8 (information shared with and agreed upon by patients) showed the largest improvement on completeness. This major improvement could be the result of the difference between retrospective reporting in the survey data collection method and prospective reporting in EHR data. In the surveys, physical therapists were asked to reflect on patient cases that were already closed. The communication process with the patient might be harder to remember for the physical therapist than the steps in clinical reasoning, resulting in more missing values on this indicator. However, EHRs are normally completed during or right after the consult with the patient, making it easier to answer questions on the communication process with the patient.

EHRs can serve as a technological checklist for clinical reasoning, as Salazar et al. also states [16]. Although this might not improve the outcomes on clinical conditions, [17] it might help prevent mistakes in the clinical process and increase safety as a relation was found between clinical incidents and poor reporting [18]. At the least it can help mistakes be more easily retrieved, increasing transparency and accountability.

One of the reasons behind the relatively small differences between indicator scores in survey data and EHR data could be the presence of ceiling effects. Ceiling effects are represented by the percentage of therapists that have the maximum indicator score. An earlier study into the psychometric properties of the survey data revealed a high level of ceiling effects [11]. This posed a problem as it was thus more difficult to distinguish between different physical therapists on the level of their quality, but also that it would be difficult to establish relevant change over time within the same therapist. This would probably explain the small changes in values when the survey data is compared to the EHR data.

Using the EHRs for quality measurements saves valuable time as clinicians do not have to complete additional surveys for quality assessments next to the regular administration of their patients. The administration is done electronically in the EHRs during or right after the therapy session and the data can be directly extracted without further action from the therapist. That is time better spent on patient care and may potentially lead to an indirect positive effect on the quality of care. EHRs present a possibility for continuous and automated data extraction for real time monitoring of the quality of care and for providing direct feedback to patients, medical professionals and health insurance companies. If software differences between the various EHRs are overcome to deliver standardized output and the process of extracting the data from the EHRs is automated, quality managers can use the quality information when it suits them instead of waiting for the results of a study or a report to act on them more promptly. Research has indicated that feedback can be used to improve the quality of care, showing improvements after feedback initiatives on process or outcome of care, although the effectiveness of feedback initiatives does depend on the (perceived) quality of the data and the willingness of the recipients [15].

### Limitations

A limitation in using EHR software was the difference in designs of the EHRs. Some of the smaller software suppliers included visual cues into their EHR design to signal the physical therapist whether or not information was missing in the patient file. Research suggests that visualization tools could have a positive effect on the number of missing values in EHRs [19]. These differences within EHR data must be overcome to ensure that physiotherapy practices can be compared on quality of care by standardizing the design, or the EHR software supplier must be controlled for in comparative research. In our study, the number of practices that used an EHR with visual clues were too small to allow for comparative analysis between EHRs.

A limitation regarding the generalizability of the findings was the overrepresentation of the group of acute care patients in the EHR data. Only data on patient intervention episodes were included that were actively closed by the therapist in the data extraction period. Given the relative short timeframe of data extraction, there is a higher chance of closing a patient intervention episode in which the patient has acute symptoms than a patient case of a patient with chronic symptoms. In the survey data, physical therapists selected from cases that were already closed, so they could select any case, including chronic cases that was closed in the last year. An earlier study confirmed that the steps in the process of clinical reasoning were significantly better followed in acute cases on half of the quality indicators, when controlling for other patient characteristics [11]. With the overrepresentation of acute patient cases, the indicator scores could be overestimated in the EHR data. However, on the quality indicators that were compared in this paper, no significant differences in scores between acute and chronic disease patients were found in the survey data for three indicators (indicators 1,2 and 3) [11] whereas on indicator 8 chronic disease patients were significantly better informed than acute patients [11]. To ensure that the differences found in this study were not the result of differences in any of the patient, therapist or practice characteristics, we performed additional multilevel regression analyses (see Additional file 2). This showed that even when controlling for these characteristics, the differences between indicator scores in the EHR data and the survey data were similar to the results presented in Table 5. We are therefore confident that the generalizability of the results in this paper is not limited by differences in the sample.

Another limitation is the absence of a gold standard on indicator scores for physical therapy. We assessed correctness in this paper by comparing the EHR data to the survey data, which we used as a benchmark. That is not to say that the survey data was ‘correct’. However, in the development process of the quality indicators, consensus rounds were held with all stakeholders; physical therapists, patients, insurers and the inspectorate to ensure content validity. Further, construct validity was positively assessed as well as reproducibility and interpretability [11]. The biggest problem in the indicators were the high ceiling effects, as mentioned before [11, 20]. It is also possible that since the data sources are of different time frames, but with the same physical therapists, changes in indicator scores are caused by a time effect. Physical therapists might have learned from participation in the first cohort and adapted their practice accordingly to improve the quality of care. However, since the differences between indicator scores calculated from survey data and EHR data are relatively small (or in the case of indicator 1 even negative), we consider the time effect not to be a major influence on the results.

### Implications for research and quality policy

Although data completeness [3] and correctness [3, 4] are important proxies of data quality, there are other important data properties left untouched in this study due to restraints in time or funding. Weiskopf and Weng for example argue that three dimensions of data quality are of fundamental value; that is correctness, completeness and currency, or timeliness [7]. The data in the EHRs must be representative of a patient state at the time of recording. Other properties are different aspects of reliability, validity and reproducibility of the data, as Terwee et al. [20] for example proposed. Although our study focused on the comparison of data quality, quality of care research would benefit from a closer look at other data properties to assess the added value of using the EHR as a data source for research purposes.

Another implication of this study is the need for effective natural language programming (NLP) tools. With these tools, text boxes in the EHR, e.g. for describing patient goals for example, can be analysed and used for a more content-based quality measurement. Although these tools can be successful, it requires a considerable user involvement [1]. Clinicians should collaborate in developing such tools to ensure that the right ‘dictionary’ is being used, i.e. the right professional lexicon. In a trade-off between a deeper linguistic understanding and computational efficiency, Jung et al. [21] advocates the use of simpler NLP tools to advance adoption of NLP in practice. A simpler, dictionary-based term recognition tool can be used, as these are easier to use and with more speed than more advanced NPL tools [21]. The use of text-mining tools allows clinicians to maintain a level of narrative information so that he can use his own words to describe his patient to administrate the treatment, while researchers can encode this information to measure the quality of care.

At the same time, we advice the use of standardized coding with a search function as another possibility for a more content-based quality measurement. For example, for encoding complaints the International Classification of Functioning, Disability and Health (ICF) could be used [22]. The ICF is an extensive effort of the World Health Organization (WHO) to standardize terminology and to classify problems in the human functioning. The classification covers multiple dimensions of human functioning with underlying categories in body functions, activities and participation, including internal and external factors that may influence human functioning. The use of the ICF in coding problems in functioning of patients would assist in establishing reliable comparisons for estimating the quality of care, although preliminary training is required to use the ICF correctly [23]. Complaints in physiotherapy are usually situational and activity related, and by this specific set of coding, it can help the physical therapist in deciding which intervention is needed and what outcome is achievable, and at the same time it can also help the quality research to step up to more content-based quality measurements [24]. The use of classification systems will transform the EHR from an ad-hoc extraction system for quality research, into a proactive documentation support system to improve the administration of health data upfront, as proposed by Botsis et al [1]. It should be studied if these standardizations of documentation and terminology [25, 26] will be effective in enhancing comparability and decreasing variation between EHR suppliers. Indicators 4 (defining and administrating intervention) and 7 (objectified result, by use of measurement instrument) for example could not be extracted from the EHRs because there was not enough time within the project to classify the possible interventions and the measurement instruments that could, or should be used in each case. With a classification system implemented in the EHRs, the correct data to calculate these two quality indicators from could be extracted from the EHRs.

## Conclusion

The main challenges are ensuring comparability between survey data and EHR data, as well as comparability between different EHR-systems. Collecting data from surveys is more costly both in time and money, and data quality of both methods was roughly the same so future efforts should be aimed at streamlining the use of EHR data for quality of care research. Standardization of the format of EHRs, the use of a standardized coding and exploring text mining tools require a considerable effort from the physiotherapy community, researchers and EHR developers. A standardized EHR can be used for continuous measurement of the quality of care, and for providing real-time feedback to all stakeholders. More research and testing is needed to bridge the needs of clinicians for using the EHR in practice and the needs of researchers and health insurers for using the EHR as a database for quality research.

## Additional files

Additional file 1: Description Qualiphy project. Additional information on the project “Quality Indicators for Physical Therapy”. (PDF 36 kb)
Additional file 2: Multilevel regression on 4 indicators with characteristics on three levels, i.e. patient, therapist and practice. To ensure that differences between the survey data and the EHR data in patient, therapist or practice characteristics did not influence the results, we have performed additional multilevel regression analyses on all four quality indicators (see Additional 2: Table A1). This provided us with evidence that our main conclusions did not change. Although the patient characteristics chronic vs. acute patient and direct access patient vs. referred patient did have a small significant effect on the quality indicators, controlling for these characteristics gave the same result, that is a small negative effect on the indicator score in the EHR data (reference category is the survey data) on indicators 1 and 2 and a small positive effect in the EHR data on indicator 3. There was no significant difference between EHR data and survey data on indicator 8, when we controlled for the patient, therapist and practice characteristics. These results are equal to the results in Table 4. (PDF 75 kb)

## Abbreviations

EHR: Electronic health record
ICF: International classification of functioning, disability and health
KNGF: Royal dutch society for physical therapy
NLP: Natural language programming
QI: Quality indicator
Qualiphy Project: Quality Indicators for Physical therapy
