# Supplementary material for: Data extraction from electronic health records (EHRs) for quality measurement of the physical therapy process: comparison between EHR data and survey data
Source: BMC Med Inform Decis Mak. 2016 Nov 8;16:141. doi: 10.1186/s12911-016-0382-4 (PMC5101697; doi:10.1186/s12911-016-0382-4)
Supplement: Additional file 1: — Description Qualiphy project. Additional information on the project “Quality Indicators for Physical Therapy”. (PDF 36 kb) [file 12911_2016_382_MOESM1_ESM.pdf]

Description Qualiphy project

For the Qualiphy project (quality indicators for physical therapy in primary care) 23 quality indicators were developed in consensus meetings among the Royal Dutch Society for Physical Therapy (KNGF), the national agent of Health Insurance Companies, the Dutch Patient and Consumer Federation, and the Healthcare Inspectorate. The set described quality of care in three domains: (1) physical therapy care process (8 indicators), (2) practice management (5 indicators), and (3) patient experiences (10 indicators). Qualiphy collected data for the calculation of the quality indicators in 2009, 2010 and 2011 and was set up as a national audit and feedback system in physical therapy. In the first two years the first domain, the physical therapy care process, was evaluated in surveys by physical therapists. In 2011 the data for this domain were extracted directly from the Electronic Health Records (EHRs). The validity and reliability of the physical therapy care process was assessed by Scholte et al.[11]. The main issue of the indicators was the presence of ceiling effects in all indicators. This made the distinction between high and low scoring therapists and practices more difficult.
