# Supplementary material for: Data extraction from electronic health records (EHRs) for quality measurement of the physical therapy process: comparison between EHR data and survey data
Source: BMC Med Inform Decis Mak. 2016 Nov 8;16:141. doi: 10.1186/s12911-016-0382-4 (PMC5101697; doi:10.1186/s12911-016-0382-4)
Supplement: Additional file 2: — Multilevel regression on 4 indicators with characteristics on three levels, i.e. patient, therapist and practice. To ensure that differences between the survey data and the EHR data in patient, therapist or practice characteristics did not influence the results, we have performed additional multilevel regression analyses on all four quality indicators (see Additional 2: Table A1). This provided us with evidence that our main conclusions did not change. Although the patient characteristics chronic vs. acute patient and direct access patient vs. referred patient did have a small significant effect on the quality indicators, controlling for these characteristics gave the same result, that is a small negative effect on the indicator score in the EHR data (reference category is the survey data) on indicators 1 and 2 and a small positive effect in the EHR data on indicator 3. There was no significant difference between EHR data and survey data on indicator 8, when we controlled for the patient, therapist and practice characteristics. These results are equal to the results in Table 4. (PDF 75 kb) [file 12911_2016_382_MOESM2_ESM.pdf]

Table A1. Multilevel regression on 4 indicators with characteristics on three levels, i.e. patient, therapist and practice

	Indicator 1	Indicator 2	Indicator 3	Indicator 8
**Patient characteristics:**				
Chronic patient (ref. acute patient)	-0.004 (0.003)	-0.001 (0.001)	**0.004 (0.001)**	**0.011 (0.004)**
Direct access patient (ref. referred patient)	n.a.*	n.a.*	**-0.005 (0.001)**	**-0.038 (0.003)**
Male (ref. female)	-0.000 (0.001)	-0.000 (0.001)	-0.001 (0.001)	-0.000 (0.002)
Age	0.000 (0.000)	-0.000 (0.000)	-0.000 (0.000)	0.000 (0.000)
EHR (ref. survey)	**-0.024 (0.002)**	**-0.035 (0.001)**	**0.022 (0.001)**	-0.003 (0.003)
**Therapist characteristics:**				
Male (ref. female)	-0.002 (0.002)	0.000 (0.002)	-0.004 (0.003)	-0.003 (0.005)
Age	-0.001 (0.000)	-0.000 (0.000)	0.000 (0.000)	0.000 (0.000)
**Practice characteristics:**				
Practice size >1 (ref. practice size =1)	0.000 (0.000)	-0.000 (0.000)	0.000 (0.000)	0.000 (0.000)

*n.a. = not applicable as indicator 1 is only for direct access patients and indicator 2 is only for referred patients

Bold: p<0.001
